# Clinical features, treatment and outcomes in patients with tracheal adenoid cystic carcinoma: a systematic literature review

**DOI:** 10.1186/s13014-021-01770-0

**Published:** 2021-02-19

**Authors:** Juntao Ran, Guofeng Qu, Xiaohua Chen, Da Zhao

**Affiliations:** Department of Radiation Oncology, The First Hospital of Lanzhou University, Lanzhou, 730000 People’s Republic of China

**Keywords:** Adenoid cystic carcinoma, Tracheal carcinoma, Systematic review

## Abstract

**Background:**

Primary tracheal adenoid cystic carcinoma (TACC) is rare and originates from the minor salivary gland. Biologically, TACC results in delayed presentation, and the therapeutic effects of multimodal treatment differ across individuals. This study aimed to review cases of TACC to identify clinical features, imaging modalities, treatment, and patient outcomes across follow-ups.

**Methods:**

The PubMed, Web of Science and MEDLINE databases were searched to identify articles reporting cases of TACC. The study variables included in the analysis were patient demographics, biological characteristics, presenting symptoms, imaging modalities, treatments, follow-up times and survival outcomes.

**Results:**

A total of 76 articles and 1252 cases were included in this review. The most common presenting symptom was dyspnoea (86.0%), followed by cough (58.0%). Surgery alone (40.9%), surgery with postoperative radiotherapy (36.4%) and radiotherapy alone (19.2%) were used most frequently treatments modalities. Of the 1129 cases with disease control and survival data, there was no evidence of disease in 78.7%, local recurrence was reported in 3.8%. Distant metastasis rate was 24.9% of 418 reported cases, lung (44.2%) was the most commonly involved organ. The 5, 10 years survival rate of patients treated with surgery alone and surgery with postoperative radiotherapy were 86.4%, 55.6% and 97.3%, 44.4%, respectively.

**Conclusion:**

TACC most common presenting symptoms were dyspnoea, cough and shortness of breath. Surgery alone and surgery with postoperative radiotherapy are predominant treatment modalities. Both seems to provide a good result in term of disease control and long-term survival rate in patients with TACC.

## Introduction

The incidence of primary tracheal carcinoma is 0.1 to 0.26 per 1,00,000 persons, accounting for 0.1–0.4% of all patients with malignant diseases [1–3]. Adenoid cystic carcinoma (ACC) is a rare malignant tumour of the salivary glands and was first described by Billroth in 1856 [4, 5]. Primary tracheal adenoid cystic carcinoma (TACC) is the second most common tracheal malignant tumour after squamous cell carcinoma [6] and accounts for approximately 10–20% of all tracheal malignant tumour cases [5, 7, 8]. In terms of its biological characteristics, TACC arises from the submucosal glands, it has an indolent course, it is characterized by a slow growth rate and late distant metastasis [1, 4]. As a result of the submucosal location of the tumours, the clinical symptoms present late. Patients with this type of tumour commonly present with dyspnoea, cough, wheezing and haemoptysis [9].

Surgical resection is the common modality of treatment for TACC, but complete resection is achieved in only 42–57% of cases [6, 10, 11]. Adjuvant radiotherapy is generally approved for TACC that were not completely excised [12–14], however, the value of adjuvant radiotherapy for TACC remains controversial according to published literatures. In patients with cases deemed unresectable or contraindications to surgery, definitive radiotherapy is proposed [6]. Only a limited number of cases have been reported, making it difficult to develop protocols based on evidence-based treatment regimens for TACC. Here, we present a systematic review of the reported cases of TACC to identify trends in demographics, presenting symptoms, treatment modalities, and outcomes.

## Materials and methods

### Search strategy

A comprehensive search of the PubMed, Web of Science and MEDLINE databases was performed to identify articles published up until December 2019, using the terms ‘‘tracheal adenoid cystic carcinoma’’, ‘‘tracheal tumours’’ and ‘‘tracheal neoplasm’.’ The results were limited to English-language articles. The title and abstract of studies identified in the search were scanned to exclude any studies that were clearly irrelevant. The full texts of the remaining articles were retrieved and read to determine whether they met the inclusion criteria. The references of the full-text articles were reviewed to identify additional relevant articles. When there was uncertainty about the eligibility of a paper, this was discussed and resolved by consensus within authors.

### Selection criteria

Studies that reported data on the diagnosis and treatment of patients with TACC were included. The exclusion criteria were articles not published in English, histology and radiologic studies, and those on other anatomic locations. Additionally, studies for which the full text could not be obtained and with insufficient data were excluded. Discrepancies among authors on article inclusion were resolved through discussion with other authors. The strength of the evidence of the included articles was assessed with the Oxford Centre for Evidence-Based Medicine classification system.

### Data extraction

Two investigators (J. Ran, G. Qu) reviewed studies and extracted the information on author, publication year, study type, sample size, demographics, presenting symptoms, imaging modalities, treatments, follow-up time, recurrence, and outcomes using a standardized form.

All the analyses were done using Microsoft Excel (2016).

## Results

### Literature search

**Fig. 1:**
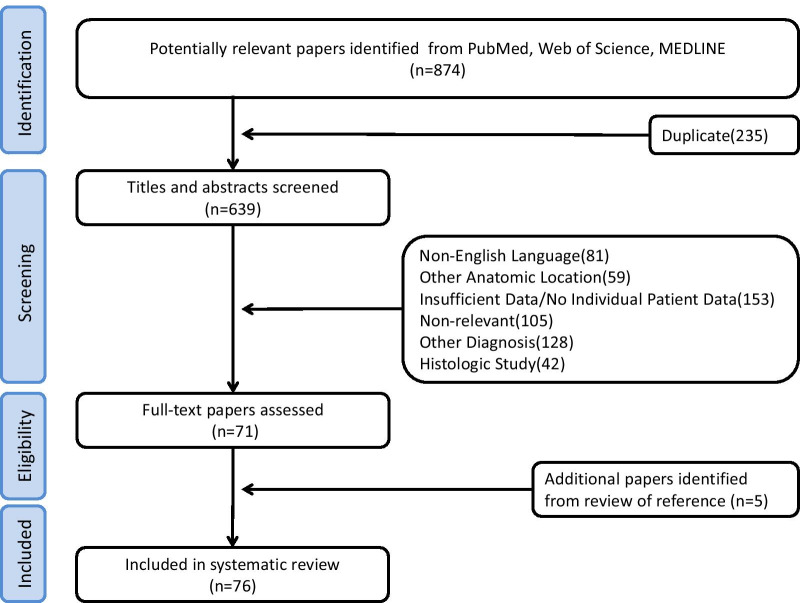
Flowchart of literature search and review

The literature search of PubMed, Web of Science, and MEDLINE identified 874 articles (Fig. 1). Among them, 803 were excluded because they failed to meet the eligibility criteria. Of the remaining 71 articles, the full texts were retrieved and the data analysed. Additionally, 5 articles were retrieved through manual searches of the reference lists. Finally, a total of 26 case series and 50 case reports, 1252 cases, were included in this review (Additional file 1: Table S1), with an aggregate level of evidence of 3b.

### Demographics and clinical characteristics

Among all 1252 patients, demographics were reported for 1088 patients, the age but not the sex of 1 patient was reported, and age was not reported for 16 patients. The average patient age was 48.7 years. The gender distribution was skewed towards females with a male to female ratio of 1:1.17. The presenting symptoms were reported for 813 cases, the most common presenting symptom was dyspnoea (86.0%), followed by cough (58.0%). The average time between the onset of symptoms and diagnosis was reported for 249 cases and was 12.5 months. The average tumour size was reported for 719 cases and was 3.25 cm. Information regarding treatment modality was available in 1239 patients. The most common primary treatment was surgery alone (40.9%) and surgery with adjuvant radiotherapy (36.4%). The average and median doses of adjuvant radiotherapy were 55.8 Gy and 54 Gy (Range: 40 Gy-77 Gy). The average and median doses of radical radiotherapy were 63.9 Gy and 60 Gy (Range: 46 Gy-80 Gy). One patient underwent stent implantation and one patient receive no treatment. Clinical characteristics are summarized in Table 1.

**Table 1:** Patient clinical characteristics and treatments

Patient characteristics	n (%)
All patients	1252
Demographics	1088
Average age (years)	48.7(14–97)
Gender	
Male	501
Female	587
Presenting symptoms	813
Dyspnea	699 (86.0)
Cough	471 (58.0)
Shortness of breath	302 (37.1)
Hemoptysis	125 (15.4)
Wheeze	32 (3.9)
Pectoralgia	20 (2.5)
Stridor	7 (0.9)
Neck swelling	3 (0.4)
Hoarseness	2 (0.2)
Radiography, Endoscopy	949
CT and Bronchoscopy	687 (72.4)
Bronchoscopy	162 (17.1)
CT or PET-CT	72 (7.6)
X-ray and Bronchoscopy	22 (2.3)
CT and larynoscopy	4 (0.4)
MRI and/or larynoscopy	2 (0.2)
Treatment modalities	1239
Surgery	507 (40.9)
Surgery and RT	451 (36.4)
RT	238 (19.2)
Surgery, RT and Chemotherapy	26 (2.1)
Surgery and Chemotherapy	10 (0.8)
RT and Chemotherapy	4 (0.03)
Chemotherapy	1 (0.01)
Other	2 (0.02)

### Radiography and endoscopy

The radiographic imaging modalities reported of 949 patients are listed in Table 1. The following imaging modalities were used alone or in combination with other techniques: computed tomography (CT), bronchoscopy, laryngoscopy, MRI, PET-CT and X-ray. The most commonly used radiographic imaging modalities were CT with bronchoscopy.

### Survival outcomes

The 1129 cases for which the follow-up time and outcome measures were available, the average follow-up time was 49.8 months, with a range from 1 to 162 months, there were no evidence of disease in 889 cases (78.7%) and local recurrence in 43 cases (3.8%). Of 418 reported cases with regional and distant metastasis data, lymph node metastasis was reported in 93 cases (22.2%), and distant metastasis in 104 cases (24.9%), and the lung (46/104, 44.2%) was the most commonly involved organ.

Survival outcomes of the different treatment modality are displayed in Table 2. At the time of this analysis, outcomes of 239 cases treated with surgery alone were available, the follow-up time was 119.4 months, 86.4% (184/213) of the patient were alive at 5 years, 55.6% (90/162) of the patients were alive at 10 years. The follow-up duration was available for 189 cases treated with surgery and postoperative radiotherapy and was 77.3 months, the 5 years and 10 years survival rate were 97.3% and 44.4% of 189 cases, respectively. The follow-up time of 58 cases treated with radiotherapy alone was 45.4 months, the 5 years and 10 years survival rate were 34.9% and 16.1% of 43 cases, respectively. Twenty-six cases were treated by surgery, radiotherapy and chemotherapy, the 5 years survival rate was 88.9% of 9 cases, and the follow-up time for 12 cases was 94.4 months.

**Table 2:** Outcomes stratified by the treatment modality used for tracheal adenoid cystic carcinoma

Treatment modality	Number of cases	Follow-up cases	Follow-up time (m)	5 years		10 years	
				Follow-up cases	survival rate, %	Follow-up cases	survival rate, %
Surgery alone	507	239	119.4	213	86.4	162	55.6
Surgery and RT	451	189	77.3	189	97.3	189	44.4
RT alone	238	58	45.4	43	34.9	43	16.1
Surgery, RT and CT	26	12	94.4	9	88.9	-	-

*RT* radiotherapy, *CT* chemotherapy

## Discussion

Most of the information about TACC is derived from case reports and short series due to the rarity of the disease, which is often restricted by limited information about treatment and survival. The present systematic review aimed to provide more comprehensive information on clinical features, patterns of treatments and survival outcomes of TACC.

The present analysis showed that the average age of the entire cohort was 48.7 years, this is comparable with literature results [10, 14–16]. Earlier studies [3, 10, 17] reported there were no significant difference between the frequency of male and female patients, which is consistent with the finding of the present analysis. Besides, our review found that the interval between the onset of symptoms and diagnosis was 12.5 months, which is similar to that reported in the majority of case series and differs from that reported in the case reports [10, 18–20]. The relatively long time may relate to the indolent clinical behaviour and non-specific sign of TACC. Furthermore, this study indicated that the most common symptom of TACC was dyspnoea, followed by cough. However, a variety of other symptoms were reported, including haemoptysis, wheezing, pectoralgia, stridor, neck swelling, and hoarseness [7, 21–24]. The discrepancy among reports may be due to the small sample sizes of most studies.

The pre-treatment imaging modality used commonly in the evaluation of TACC was CT and bronchoscopy [18, 25]. In addition, MRI, PET-CT, X-ray and laryngoscopy were also used for examinations [22, 26–28]. According to our assessment of radiologic modality, the most common modalities performed to establish the diagnosis and assess the condition of the disease were also CT and Bronchoscopy. CT imaging can be used to determine the tumour location, extension, regional invasion and lung metastasis, and provide important information for treatment options. Bronchoscopy reveals the location and growth of the tumour, and biopsy can be performed for a pathological diagnosis. MRI and PET-CT were adopted in relatively little cases, the manifestation and value of them in diagnosis of TACC need to further evaluate. But PET-CT may have a role for residual tumour and target delineation of radiotherapy.

It is wildly accepted that radical surgical resection is associated with better survival outcomes in TACC. Studies have reported that the 5-year survival rate was 88%-100%, the 10-year survival rate was 51%-80% with surgery alone [14, 17, 18, 29]. These results are comparable with the survival rate in this review (5-year survival rate 86.4%; 10-year survival rate 55.6%). However, given the limited scope of tracheal resection and the tendency for TACC to spread in submucosal tissue, the complete resection is not always achievable, the positive rate of resection margin is 59–63%. In addition, the extent of excision can increase the surgical complication and operative mortality rates [21]. Some studies reported that the mortality rate related to surgical procedures was 1%-25% [14, 21, 22, 30]. Hence, postoperative radiotherapy is usually recommended for TACC with non-R0 resection. Surgical margin status and palliative resection were identified to be associated with receiving adjuvant radiotherapy in routine clinical practice, and it can increase the survival rate and decrease the risk of local recurrence and distant metastasis [16, 31–33]. But the inclusion of radiotherapy in the treatment of TACC remain controversial because of no trials exploring the role of postoperative radiotherapy [2, 14, 30, 34, 35]. In the review, surgery and postoperative radiotherapy was used in 36.4% of patients, and the 5-year survival rate was 97.3%, the 10-year survival rate was 44.4%. This clearly supports the role of postoperative radiotherapy in TACC.

For unresectable disease, definitive radiotherapy is generally employed. Nevertheless, very little reports have been indicated on nonsurgical therapeutic alternatives. In our review, 34.9% patient lived more than 5 years, 16.1% more than 10 years after radiotherapy alone. Although this survival rate is inferior to surgery alone and postoperative radiotherapy, it is obviously that radiotherapy is a good therapeutic alternative for unresectable TACC. Furthermore, Högerle et al. [29] reported that the curative effect of definitive radiotherapy is similar to that of surgery with radiotherapy.

Several limitations should be considered when interpreting these results. First, the quality of all the included publications represents primary limitation in the review. The studies did not uniformly report variables of observational indexes and information regarding symptoms, tumour size, imaging modalities, treatment modalities and survival rate was incomplete in most reports. Due to the heterogeneity of the studies and the different follow-up times of the subgroups, the analysis is susceptible to bias. Second, according to the Oxford Centre for Evidence-based Medicine, the majority of studies included in this review had evidence levels of 3 and 4, indicating poor levels of evidence. But being a rare tumour very little data is available, it is difficult to get good quality data and a randomized controlled trial is very difficult to conduct. Finally, the studies in this systematic review were published from 1969 to 2019, the management of technology and treatment is not homogeneous and may influence outcome results during this time. Hence, our results may underestimate benefit of modern technology of surgery and radiotherapy. However, though not conclusive, our finding will provide directions to physicians to make treatment decisions till better quality data comes up.

## Conclusion

TACC commonly presents with dyspnoea and cough. The main examination modalities were CT and bronchoscopy. Treatment of TACC is challenging, surgery alone and surgery followed by postoperative radiotherapy were the most performed treatment modalities, and the long-term survival rate was high in the majority of patients at the follow-up. In patients presenting with an unresectable/inoperable tumour, definitive radiotherapy is recommended as appropriate therapy.

## Supplementary Information

**Additional file 1**. Studies meeting criteria for the systematic review.

## Data Availability

All data generated or analysed during this study are included in this published article.

## Abbreviations

ACC: Adenoid cystic carcinoma
RT: Radiotherapy
CT: Chemotherapy
BS: Bronchoscopy
LS: Larynoscopy
OS: Overall survive
