# Supplementary material for: Clinical features, treatment and outcomes in patients with tracheal adenoid cystic carcinoma: a systematic literature review
Source: Radiat Oncol. 2021 Feb 19;16:38. doi: 10.1186/s13014-021-01770-0 (PMC7893857; doi:10.1186/s13014-021-01770-0)
Supplement: Supplementary file 1 — Additional file 1. Studies meeting criteria for the systematic review. [file 13014_2021_1770_MOESM1_ESM.docx]

**Table S1**. Studies Meeting Criteria for Systematic Review.

| Author | Year | Study Type | Cases, n | Level of Evidence^a^ |
| --- | --- | --- | --- | --- |
| Abike^1^  Al Khatib^2^  Aldrees^3^  Allen^4^  Alongi^5^  Archuna^6^  Azar^7^  Baekelandt^8^  Baydur^9^  Bonner^10^  Bots^11^  Calzada^12^  Chan^13^  Chang^14^  Charlton^15^  Chen^16^  Cheung^17^  Choudhury^18^  Chowdhary^19^  Das^20^  Elktaibi^21^  Gaissert^22^  Gupta^23^  Haresh^24^  Hashimoto^25^  Hassan^26^  He^27^  Honings^28^  Hogerle^29^  Hsu^30^  Huo^31^  Je^32^  Jeffrey^33^  Kim^34^  Kobayashi^35^  Kokturk^36^  Kukwa^37^  Levy^38^  Li^39^  Marjany^40^  Masih^41^  Maziak^42^  Nakanishi^43^  Nakratzas^44^  Kohno^45^  Nicolini^46^  Ning^47^  Nomori^48^  Nuwal^49^  Okahara^50^  Pawlewicz^51^  Pearson^52^  Pei^53^  Prommegger^54^  Qi^55^  Rajat^56^  Shafiee^57^  Santhosh^58^  Shadmehr^59^  Spatola^60^  Spinelli^61^  Stalpaert^62^  Sweeney^63^  Subramaniam^64^  Tajima^65^  Varghese^66^  Verma^67^  Wang H^68^  Wang D^69^  Wo^70^  Wright^71^  Yang P^72^  Yang H^73^  Yasumatsu^74^ Zhao^75^  Zunker^76^ | 2011  2017  2016  2007  2008  2000  1998  1989  1975  2012  2019  2012  2014  2011  2015  2015  1989  2013  2019  2017  2015  2004  2016  2008  2018  2015  2016  2010  2019  2015  2014  2017  2019  2013  1995  2004  2014  2017  2012  2014  2010  1996  2005  1974  1995  2019  2019  2014  2019  1996  2018  1974  2019  1998  2016  2016  2018  2016  2011  2019  2019  1979  2016  2019  2015  2017  2018  2017  2018  2018  1996  2005  2016  2012  2013  1969 | Case report  Case report  Case report  Case report  Case report  Case report  Case report  Case series  Case report  Case report  Case report  Case series  Case report  Case report  Case report  Case report  Case series  Case report  Case report  Case report  Case report  Case series  Case report  Case report  Case report  Case report  Case report  Case series  Case series  Case series  Case series  Case series  Case series  Case report  Case report  Case report  Case report  Case series  Case report  Case series  Case report  Case series  Case report  Case report  Case report  Case report  Case series  Case report  Case report  Case report  Case report  Case series  Case series  Case series  Case report  Case report  Case report  Case series  Case series  Case series  Case report  Case report  Case report  Case report  Case report  Case report  Case report  Case report  Case report  Case series  Case report  Case series  Case series  Case report  Case series  Case report | 1  1  1  1  1  1  6  2  1  1  1  5  1  1  1  56  4  1  1  1  1  135  1  1  1  1  1  108  38  33  21  22  132  1  1  1  1  31  1  2  1  38  1  1  1  1  42  1  1  1  1  16  7  16  1  1  1  6  17  4  1  1  1  1  1  1  1  1  1  263  1  7  109  1  82  1 | 5  5  5  5  5  5  4  4  5  5  5  4  5  5  5  4  4  5  5  5  5  4  5  5  5  5  5  4  4  4  4  4  4  5  5  5  5  4  5  4  5  4  5  5  5  5  4  5  5  5  5  4  4  4  5  5  5  4  4  4  5  5  5  5  5  5  5  5  5  4  5  4  4  5  4  5 |

^a^ Per the Oxford Centre for Evidence-Based Medicine classification system

**Reference**

1. Abike F, Bingol B, Temizkan O, Dunder I, Kilic GS, Cetin G, et al. Primary tracheal adenoid cystic carcinoma and tracheal tumors during pregnancy. Rare Tumors.2011;3:104-107.
2. Al Khatib S, Asha W, Khzouz O, Barakat F, Khader J. Adenoid cystic carcinoma with thyroid invasion mimicking thyroid cancer treated with definitive radiation: Case report and review of the literature.Case Rep Oncol.2017;10:706-712.
3. Aldrees T, Alanazi A, Fatani H, Samman A, Aldhahri SF. Adenoid cystic carcinoma of the upper airway mimicking a thyroid tumor: A case report. Molecular and Clinical Oncology.2016;5(2):367-370.
4. Allen AM, Rabin MS, Reilly JJ, Mentzer S. Unresectable adenoid cystic carcinoma of the trachea treated with Chemoradiation. Diagnosis in Oncology.2007;13:5521-5523.
5. Alongi F, Muzio DN, Motta M.Adenoid cystic carcinoma of trachea treated with adjuvant hypofractionated tomotherapy. Case report and literature review. Tumori.2008;94:121-125.
6. Archuna U. Adenoid cystic carcinoma of the trachea: a case report. Med J Malaysia 2000; 55:273-276.
7. Azar T, Abdul-Karim FW, Tucker HM. Adenoid cystic carcinoma of the trachea. Laryngoscope.1998;108:1297-1300.
8. Baekelandt BM, Verschakelen JA, Baert AI.Primary tracheal and bronchial adenoid cystic carcinoma(Cylindroma).Fortschr Rontgenstr.1989;151:381-383.
9. Baydur A, Gottlieb L. Adenoid Cystic Carcinoma (Cylindroma) of the Trachea Masquerading as Asthma. JAMA 1975; 234:829-831.
10. Bonner LP, Stripp D, Cooper JD, Both S, James P, Rengan R. Definitive radiotherapy for unresected adenoid cystic carcinoma of the trachea. Chest.2012;141(5):1323-1326.
11. Bots EMT,Van Wyk AC, Janson JT, Wagenaar R, Paris G, Koegelenberg CFN,et al.Syncope due to tracheal adenoid cystic carcinoma. Respirology Case Report.2019;7(7):1-3.
12. Calzada AP, Miller M, Lai CK, Elashoff DA, Abemayor E, John MAS. Adenoid cystic carcinoma of the airway: a 30-year review at one institution. Am J Otolaryngol 2012; 33:226-231.
13. Chan WL, Lee VHF, Siu SWK, Leung TW. Inoperable Adenoid Cystic Carcinoma of Trachea: Complete Remission after Multi-modality Treatment. Hong Kong Journal of Radiology 2014:203-207.
14. Chang CY, Cheng SL, Chang SC. Adenoid cystic carcinoma of trachea treated with tumor curettage and adjuvant intensity modulated radiation therapy. Southern Medical Journal.2011;104:68-70.
15. Charlton P, Pitkin L. Airway compromise due to adenoid cystic carcinoma obstructing the distal trachea: a review of current management and clinical trails. BMJ Case Rep.2015;10:1-3.
16. Chen F, Huang M, Xu Y, Li T, Xie K,Zhang L, *et al.* Primary tracheal adenoid cystic carcinoma: adjuvant treatment outcome. Int J Clin Oncol 2015; 20:686-692.
17. CheungAY. Radiotherapy for primary carcinoma of the trachea. Radio and Oncol.1989;14:279-285.
18. Choudhury BK, Barman G, Singh S, Ahmed K*.* Adenoid Cystic Carcinoma of the Upper Trachea: A Rare Neoplasm. Journal of Clinical Imaging Science.2013;3:1-4.
19. Chowdhary RL, Chufal KS, Pahuja AK. Image-guided volumetric modulated arc therapy (IG-VMAT) for unresectable ACC of the trachea: a feasible curative option. BMJ Case Rep.2019;12:1-7.
20. Das S, Yogaraja D, Anandakrishnan K. Adaptive radiotherapy in a case of adenoid cystic carcinoma of bronchus and its favourable impact on treatment outcome: A case report. Report of Practical Oncology and Radiotherapy.2017;22:282-284.
21. Elktaibi A，Elhammoumi M, Boudhas A, Arsalane A, Eloueriachi F, Oukabli M, et al. Adenoid cystic carcinoma of the trachea: a clinico-pathological analysis. Pan African Medical Journal.2015;20:1-5.
22. Gaissert HA, Grillo HC, Shadmehr MB, Wright CD, Gokhale M, Wain JC, *et al.* Long-Term Survival After Resection of Primary Adenoid Cystic and Squamous Cell Carcinoma of the Trachea and Carina. The Annals of Thoracic Surgery 2004; 78:1889-1897.
23. Gupta D, Singh I, Sakthivel P. Adenoid Cystic Carcinoma of Trachea: A Diagnostic and Therapeutic Challenge. Indian J Otolaryngol Head Neck Surg.2016;68(1):94-96.
24. Haresh KP, Prabhakar R, Rath GK, et al. Adenoid cystic carcinoma of the trachea treated with PET-CT based intensity modulated radiotherapy. Journal of Thoracic Oncology.2008;3(7):793-795.
25. Hashimoto S, Sumida Y, Tobinaga S, Wada H, Wakata K, Nonaka T,et al. Liver resection for metastasis of tracheal adenoid cystic carcinoma: Report of two cases. International Journal of Surgery Case Report.2018;48:26-29.
26. Hassan M, Quraeshi S, Zubairi AB. A rare cause of recurrent wheeze and seizures. BMJ Case Rep.2015;21:1-3.
27. He JX, Wang W, Li JP, Yin QW. Video-assisted thoracoscopic surgery tracheal resection and carinal reconstruction for tracheal adenoid cystic carcinoma. J Thorac Dis.2016;8(1):198-203.
28. Honings J, Gaissert HA, Weinberg AC, Mark EJ, Wright CD, Wain JC, *et al.* Prognostic value of pathologic characteristics and resection margins in tracheal adenoid cystic carcinoma. European Journal of Cardio-Thoracic Surgery 2010; 37:1438-1444.
29. Högerle BA, Lasitschka F, Muley T, Bougatf N, Herfarth K, Adeberg S, *et al.* Primary adenoid cystic carcinoma of the trachea: clinical outcome of 38 patients after interdisciplinary treatment in a single institution. Radiation Oncology 2019; 14(117):1-9.
30. Je HU, Song SY, Kim DK, Kim YH, Jeong SY, Back GM, *et al*. A 10-year clinical outcome of radiotherapy as an adjuvant or definitive treatment for primary tracheal adenoid cystic carcinoma. Radiation Oncology2017;12(1):196.
31. Huo Z, Wu H, Li S, Liang Z. Molecular genetic studies on EGFR, KRAS, BRAF, ALK, PIK3CA, PDGFRA, and DDR2 in primary pulmonary adenoid cystic carcinoma. Diagn Pathol 2015; 10:161.
32. Hsu AA, Tan EH, Takano AM. Lower Respiratory Tract Adenoid Cystic Carcinoma: Its Management in the Past Decades. Clin Oncol (R Coll Radiol) 2015; 27:732-740.
33. JeffreyYang CF, Shah SA, Ramakrishnan D, Raman V, Diao K, Wang H, *et al.* Impact of Positive Margins and Radiation After Tracheal Adenoid Cystic Carcinoma Resection on Survival. Ann Thorac Surg 2020; 109:1026-1032.
34. Kim D, Hwang YI, Choi S, Park C, Lee N, Kim EA, *et al.* A case of tracheal adenoid cystic carcinoma in a worker exposed to rubber fumes. Ann Occup Environ Med 2013;17:25(1):22.
35. Kobayashi T, Konaka C, Shibanuma H, Serizawa H, Ebihara Y, Kato H. A unique case of adenoid cystic carcinoma of the left main bronchus. Diagnostic and Therapeutic Endoscopy.1995;1:237-240.
36. Kokturk N, Demircan S, Kurul C, Turktas H.Tracheal adenoid cystic carcinoma masquerading asthma: A case report. BMC Pulmonary Medicine.2004;4(10):1-4.
37. Kukwa W, Korzen P, Wojtowicz P, Sobczyk G, Kiprian D, Kawechi A, et al. Tracheal adenoid cystic carcinoma mimicking a thyroid tumor: A case report. Oncology Letters.2014; 8(3):1312-1316.
38. Levy A, Omeiri A, Fadel E, Le Pechoux C. Radiotherapy for Tracheal-Bronchial Cystic Adenoid Carcinomas. Clin Oncol (R Coll Radiol) 2018; 30:39-46.
39. Li W, Hua W, Yan FG. Adenoid cystic carcinoma of trachea: a case report and review of literature. Chin Med J.2012;125(12):2238-2239.
40. Marjany ME, Arsalane A, Sifat H, Andaloussi K, Oukabli M, Hadadi K, et al. Primary adenoid cystic carcinoma of the trachea: a report of two cases and literature review. Pan African Medical Journal. 2014;19:32(1-5).
41. Masih I, Porter G, Porter S, Clarke R, Sidhu p, Harney J, *et al.* Primary adenoid cystic carcinoma of the bronchus in a female teenager. BMJ Case Rep 2010; 8:1-4.
42. Maziak DE. Biology of Adenoid Cystic Carcinoma of the Tracheobronchial Tree and Principles of Management. Thoracic Surgery Clinics 2018; 28:145-148.
43. Nakanishi K, Kuruma T. Video-assisted thoracic tracheoplasty for adenoid cystic carcinoma of the mediastinal trachea.Surgery.2005:137(2):250-252.
44. Nakratzas G, Wagenaar JPM, Reintjes M, Scheffer E, Swierenga J. Repeated partial endoscopic resection as treatment for two patients with inoperable tracheal tumours. Thorax.1974;29:125-131.
45. Kohno N, Tateno H, Kawaida M, Fukuda H. Primary Adenoid Cystic Carcinoma of the Trachea: A Case Report of a Twelve Year Survivor. Keio J Med.1995;44 (1): 30-32.
46. Nicolini EM, Montessi J, Vieira JP, Rodrigues GA, Costa VO, Teixeira FM, et al. Adenoid Cystic Carcinoma of the Trachea: A Case Report. Am J Case Rep.2019;20:1373-1377.
47. Ning Y, He W, Bian DL, Xie D, Jiang GN*.* Tracheo-bronchial adenoid cystic carcinoma: A retrospective study. Asia Pac J Clin Oncol 2019; 15:244-249.
48. Nomori H, Abe M, Sugimura H, Takeshi A. Adenoid cystic carcinoma of the lower trachea treated by resection of 11 of 18 rings of the total length: report of a case. Gen Thora Cardiovasc Surg.2016;64(4):231-233
49. Nuwal P, Dixit R, Singhal AK. Primary adenoid cystic carcinoma of trachea presenting as midline neck swelling and mimicking thyroid tumor: A case report and review of literature. Lung India 2010; 27:167-169.
50. Okahara M SY, Takigawa N,Segawa Y, Maeda Y, Takata I, Kataoka M, *et al*. Primary Adenoid Cystic Carcinoma of the Trachea Effectively Treated with the Endoscopic Nd-YAG Laser Followed by Radiation. Internal Medicine1996;35:146-149.
51. Pawlewicz K, Szutkowski Z, Kawecki A. Recurrence of adenoid cystic carcinoma of the trachea treated with radical radiotherapy: A case report. Oncol Lett 2018; 15:3890-3894.
52. Pearson FG, Thompson DW, Weissberg D, Simpson WJ, Kergin FG*.* Adenoid cystic carcinoma of the trachea. Experience with 16 patients managed by tracheal resection. Ann Thorac Surg 1974; 18:16-29.
53. Pei JM, Flieder DB, Patchefsky A, Talarchek JN. Detecting MYB and MYBL1 fusion genes in tracheobronchial adenoid cystic carcinoma by targeted RNA-Sequencing. Mod Pthol.2019;32(10):1416-1420.
54. Prommegger R, Salzer GM. Long-term results of surgery for adenoid cystic carcinoma of the trachea and bronchi. Eur J Surg Oncol 1998; 24:440-444.
55. Qi DJ, Feng L, Li J, Liu B. Primary adenoid cystic carcinoma of the trachea with thyroid invasion: A case report and literature review. Onco Targets and Therapy.2016;9:6291-6296.
56. Rajat B, Gopal MM, Mathews S. Tracheolaryngeal adenoid cystic carcinoma.Sultan Qaboos University Med J.2016;16:261-262.
57. Shafiee S, Adno A, French B, Johansson C, Frankel A, Williamson JP. Central airway obstruction caused by adenoid cystic carcinoma in pregnancy: a case report and review. Respirology Case Reports.2018;6(5):1-5.
58. Santhosh Kumar N, Iype EM, Thomas S, Sankar UV. Adenoid Cystic Carcinoma of the Trachea. Indian J Surg Oncol 2016; 7:62-66.
59. Shadmehr MB, Farzanegan R, Graili P, Javaherzadeh M, Arab M, Pejhan S, *et al.* Primary major airway tumors; management and results. Eur J Cardiothorac Surg 2011; 39:749-754.
60. Spatola C, Tocco A, Marletta D, Milazzotto R, Marletta F, Pergolizzi S, *et al.* Adenoid cystic carcinoma of trachea: long-term disease control after endoscopic surgery and radiotherapy. Future Oncol 2020;16(16S):33-39.
61. Spinelli GP, Miele E, Prete AA, Russo GL, Marzo AD, Tomao S, et al. Combined surgery and radiotherapy as curative treatment for tracheal adenoid cystic carcinoma:a case report. Journal of Medical Case Reports.2019;13(52):1-5.
62. Stalpaert G, Deneffe G, Maele RV. Surgical treatment of adenoid cystic carcinoma of the left main bronchus and trachea by left pneumonectomy,resection of 7.5 cm of trachea, and direct reanastomosis of right lung.Thorax.1979;34:554-556.
63. Sweeney L, Vernimmen F, Sinske S. MRI of a recurrent adenoid cystic carcinoma of the trachea, treated with fast neutron therapy. BJR Case Rep 2016; 2:20150201.
64. Subramaniam T, Lonnon P, Kinsella J, O’Neill JP. Laryngeal preservation in managing advanced tracheal adenoid carcinoma. Case Report in Otolaryngology.2015;2015:404586.
65. Tajima S, Koda K. Adenoid cystic carcinoma of the right main bronchus showing squamous differentiation and mimicking mucoepidermoid carcinoma:a case report. Int Clin Exp Pathol.2015;8(5):5830-5836.
66. Varghese A, Suneha S, Watkinson JC. Adenoid cystic carcinoma of Trachea. Indian J Surg.2017;79(1):67-69.
67. Verma V, Lin LY, Simone CB. Proton beam therapy for bronchogenic adenoid cystic carcinoma: Dosimetry, Toxicities, and Outcome. Int J Particle Therapy.2018;17:1-9.
68. Wang HM. Case report of apatinib mesylate treatment in rare advanced tracheal adenoid cystic carcinoma. Thoracic Cancer.2017;8:729-733.
69. Wang DQ, Bi N, Chen DF. Complete remission after hypofractionated radiotherapy for a patient with inoperable adenoid cystic carcinoma of bronchus.Medicine.2018;97:49(1-5).
70. Wo Y, Li S, Wang Y, Lu T, Qin Y, Sun X, *et al.* Predictors of nodal metastasis and prognostic significance of lymph node ratio and total lymph node count in tracheobronchial adenoid cystic carcinoma. Cancer Manag Res 2018; 10:5919-5925.
71. Wright CL, Gandhi M, Mitchell CA. Adenoid cystic carcinoma of the left main bronchus mimicking MacLeod's syndrome. Thorax 1996; 51:451-452.
72. Yang PY, Liu MS, Chen CH,et al. Adenoid cystic carcinoma of the trachea: a report of seven cases and literature review. Chang Guang Med.2005;28:357-363.
73. Yang H, Yao F, Tantai J, Zhao Y, Tan Q, Zhao H*.* Resected Tracheal Adenoid Cystic Carcinoma: Improvements in Outcome at a Single Institution. The Annals of Thoracic Surgery 2016; 101:294-300.
74. Yasumatsu R, Fukushima J, Nakashima T, Kadota H, Segawa Y, Tamae A, et al. Surgery management of malignant tumors of the trachea: Report of two cases and review of literature. Case Rep Oncol.2012;5:302-307.
75. Zhao Y, Zhao H, Fan L, Shi J. Adenoid cystic carcinoma in the bronchus behaves more aggressively than its tracheal counterpart. Ann Thorac Surg 2013; 96:1998-2004.
76. Zunker HO, Moore RL, Baker DC, Lattes R. Adenoid cystic carcinoma (cylindroma) of the trachea: case report with 9-year follow-up. Cancer 1969; 23:699-707.
